# VFGF: Virtual Frame-Augmented Guided Prediction Framework for Long-Term Egocentric Activity Forecasting

**DOI:** 10.3390/s25185644

**Published:** 2025-09-10

**Authors:** Xiangdong Long, Shuqing Wang, Yong Chen

**Affiliations:** 1 College of Computer and Information Engineering, Tianjin Normal University, Tianjin 300387, China; fmclxd@163.com; 2 Department of Biology & Biomedical Sciences, Rowan University, Glassboro, NJ 08028, USA

**Keywords:** action anticipation, egocentric vision, recurrent neural network, transformer, visual semantic fusion

## Abstract

Accurately predicting future activities in egocentric (first-person) videos is a challenging yet essential task, requiring robust object recognition and reliable forecasting of action patterns. However, the limited number of observable frames in such videos often lacks critical semantic context, making long-term predictions particularly difficult. Traditional approaches, especially those based on recurrent neural networks, tend to suffer from cumulative error propagation over extended time steps, leading to degraded performance. To address these challenges, this paper introduces a novel framework, Virtual Frame-Augmented Guided Forecasting (VFGF), designed specifically for long-term egocentric activity prediction. The VFGF framework enhances semantic continuity by generating and incorporating virtual frames into the observable sequence. These synthetic frames fill the temporal and contextual gaps caused by rapid changes in activity or environmental conditions. In addition, we propose a Feature Guidance Module that integrates anticipated activity-relevant features into the recursive prediction process, guiding the model toward more accurate and contextually coherent inferences. Extensive experiments on the EPIC-Kitchens dataset demonstrate that VFGF, with its interpolation-based temporal smoothing and feature-guided strategies, significantly improves long-term activity prediction accuracy. Specifically, VFGF achieves a state-of-the-art Top-5 accuracy of 44.11% at a 0.25 s prediction horizon. Moreover, it maintains competitive performance across a range of long-term forecasting intervals, highlighting its robustness and establishing a strong foundation for future research in egocentric activity prediction.

## 1. Introduction

Driven by emerging applications in smart surveillance, virtual reality (VR), industrial automation, and healthcare monitoring, behavior prediction from first-person perspective videos has become a cornerstone of intelligent systems [1]. Long-term Action Anticipation (LTA) leverages video sequences captured by sensors to infer future behavioral trends, thereby facilitating real-time decision-making and dynamic interactions [2]. For instance, LTA supports various applications [1,2,3,4,5,6], including the detection of anomalous behaviors in smart surveillance systems, the interpretation of user intentions in VR environments, the coordination of human–machine collaboration in industrial settings, and the analysis of patient movements in telemedicine. The emergence of large-scale egocentric video datasets [2,7,8,9,10], such as Ego4D [7] and EPIC-Kitchens [2], has provided a strong foundation for the development of algorithms, significantly enhancing model performance.

**Figure 1:**
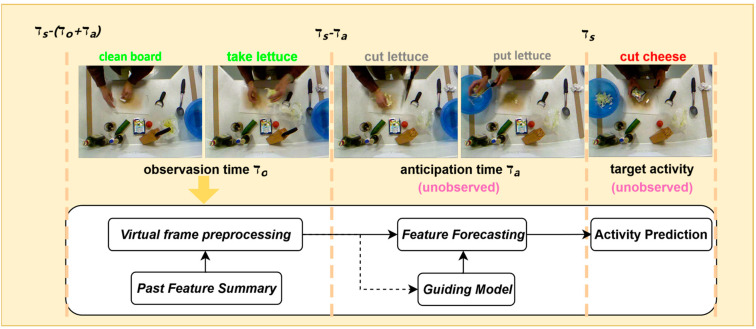
Illustration of action anticipation task and our solution.

LTA plays a pivotal role in sensor-driven intelligent systems by inferring future behavioral states from limited video sequences [2]. As illustrated in Figure 1, the LTA task involves analyzing observable video segments from an initial time $\tau_{s}-\left(\tau_{o}+\tau_{a}\right)$ to an endpoint $\tau_{s}-\tau_{a}$, aiming to predict the activity state at a future time $\tau_{s}$, where the prediction interval is defined as $\tau_{a}$, representing the unseen segment. The complexity of this task stems from multiple factors, including objective challenges such as dynamic environmental contexts and sensor-induced noise, as well as subjective factors, including varied manifestations of the same activity and diverse intentions driven by individual differences.

Despite recent progress, LTA remains a challenging task in complex, real-world scenarios, as existing models often face a trade-off between real-time responsiveness and long-term prediction accuracy. Sensor-derived video data are frequently affected by low frame rates, motion blur, or environmental noise, which result in incomplete and ambiguous semantic information [2]. The use of fixed-interval frame sampling can further exacerbate semantic loss by potentially missing critical actions. Although Recurrent Neural Networks (RNNs) [11] have shown promising performance in LTA tasks, their recursive operations result in error accumulation, which limits long-term prediction accuracy. Methods attempt to address these challenges by leveraging semantic information from observable data [12,13,14,15]. However, these approaches often heavily rely on historical observations, constraining predictions to specific patterns and making them prone to cumulative errors. Recent studies [16,17,18,19,20,21] have made progress by incorporating multimodal data or pretrained large-scale models, but these methods involve complex multimodal fusion processes, high data acquisition costs, and substantial computational resource demands.

To overcome these limitations, a Virtual Frame-Augmented Guided Prediction Framework (VFGF) is proposed. This framework aims to enhances the accuracy and efficiency of long-term action anticipation in egocentric vision tasks by addressing semantic gaps and error accumulation. The framework employs frame similarity analysis to detect significant differences between adjacent frames and generates transitional frames through linear interpolation, thereby smoothing semantic variations across activity sequences and enhancing the completeness of encoded information. Virtual frame generation is introduced to LTA tasks for the first time, addressing semantic deficiencies in observable frame sequences and providing a richer informational foundation for subsequent encoding. Built upon a Gated Recurrent Unit (GRU) [22] backbone, the Feature Guidance Module (FGM) is proposed to capture latent associations between current semantics and target activities through pretraining, dynamically generating guiding features during recursive prediction to reduce dependence on observable frames and improve the stability and robustness of long-term predictions. Extensive experiments conducted on the EPIC-Kitchens datasets validate that the proposed VFGF framework outperforms state-of-the-art methods in long-term prediction tasks, confirming its effectiveness in egocentric vision applications.

The remainder of this paper is organized as follows: Section 2 reviews related work on egocentric action recognition and video prediction. Section 3 presents the VFGF framework, detailing its virtual frame augmentation and FGM components. Section 4 describes the experimental setup and results, including comparisons with existing methods. Section 5 discusses the implications, limitations, and future directions of the proposed approach. Section 6 concludes this paper, summarizing the key findings and contributions.

## 2. Related Work

This section reviews the literature on egocentric video understanding, with a focus on identifying methodologies for action recognition and prediction. The review aims to analyze the evolution of approaches, from single-modality classification to complex multimodal fusion and long-term forecasting, and to examine how existing works address inherent challenges such as error accumulation, multimodal fusion, and the exploration of novel architectures.

### 2.1. Egocentric Action Recognition

Egocentric action recognition is a cornerstone of sensor-driven intelligent systems, focusing on analyzing and predicting human behavior patterns through egocentric video data. Recent advances in deep learning, particularly convolutional neural networks and transformer models, have significantly propelled progress in this field [23,24,25,26,27,28,29,30,31,32,33,34]. Early research on egocentric video recognition focused primarily on activity classification using visual information. For example, Liu et al. [25] introduced a video Transformer with local inductive bias by adapting the Swin Transformer from the image domain, leveraging pretrained image models to achieve an optimal balance between speed and accuracy compared to global self-attention approaches. Building on this foundation, significant progress has been made through multimodal data fusion. Xaviar [30] addressed the issues of continuous data missingness and noise in multimodal sensor data from IoT devices with the Centaur model, which combines a convolutional denoising autoencoder for data cleaning with a deep convolutional neural network incorporating self-attention for multimodal fusion. Trained with random data corruption, Centaur surpassed baseline models across multiple inertial measurement unit datasets. Similarly, Masashi et al. [31] proposed the MM-CDFSL method, which enhances target domain adaptability via multimodal distillation and employs ensemble mask inference to reduce input token counts, thereby improving inference efficiency. More recently, Wang et al. [32] addressed the labor-intensive challenge of action annotation in egocentric videos captured by wearable devices in complex backgrounds by introducing a semi-supervised learning approach based on the interaction knowledge distillation. This method utilizes a teacher-student framework, where the teacher network employs a graph neural network to capture spatiotemporal interactions between hands and objects in annotated videos, enabling the student network to learn this knowledge and improve recognition performance.

### 2.2. Egocentric Video Prediction

The release of large-scale egocentric video datasets, such as EPIC-Kitchens and Ego4D, has spurred extensive research in egocentric video prediction, leading to substantial advancements [2,12,13,14,15,16,17,18,19,20,21,33,34,35,36]. In the domain of feature learning, Furnari et al. [12]. introduced the Rolling-Unrolling LSTM architecture, which employs dual LSTMs for historical summarization and future inference, integrates sequence completion pretraining, and utilizes a modality attention mechanism to fuse multimodal features effectively. To address error accumulation in recursive sequence prediction for egocentric activity forecasting, Qi et al. [14] proposed the self-regulated learning (SRL) framework, leveraging contrastive loss to highlight information in current frames, a dynamic reweighting mechanism to capture inter-frame correlations, and multi-task learning to enhance feature representations. Liu et al. [16] addressed error accumulation and semantic deficiencies in egocentric activity prediction through the HRO hybrid framework, which combines memory-augmented recursion with one-shot representation prediction. For long-term action prediction in egocentric videos, Esteve et al. [17] developed a hierarchical architecture utilizing H3M to extract both high- and low-level human information and I-CVAE to generate stable predictions. Zhang et al. [19] focused on object-centric representations for long-term video action prediction, employing visual-language pretrained models to extract task-specific representations via “object prompts” and integrating Transformer architectures to retrieve relevant objects, with effectiveness validated across multiple datasets.

### 2.3. Summary

Research in egocentric action recognition has evolved from leveraging single visual modalities to incorporating multimodal data, aiming to enhance robustness and contextual understanding. In the closely related task of egocentric video prediction, mainstream approaches have primarily focused on two strategies: (1) designing advanced recurrent architectures to better model historical context and mitigate error propagation in recursive prediction and (2) incorporating external information, such as large-scale pre-trained models or multi-modal data, to compensate for perceptual uncertainties and semantic deficiencies in observed frames.

Despite these advancements, a significant gap remains in developing a concise and efficient framework that reduces reliance on costly external data acquisition or complex fusion processes. Many methods still face a trade-off between model complexity and generalizability, particularly in recovering missing semantic information due to sensor limitations or rapid activity transitions. To address this gap, our research introduces a solution that focuses on internal frame information augmentation and feature guidance, aiming to smooth semantic variations and reduce long-term dependency without external data.

## 3. Proposed Method

This study focuses on forecasting activity states at specific future time points by analyzing fixed-length video sequences in the context of egocentric activity prediction. Formally, given an observable video segment with time steps o, we divide and obtain frame sequence $V=\left\{v_{1},{\;v}_{2}\;,...,v_{o}\right\}$ at time interval τ, where each $v_{i}\in R^{d}$ represents the frame at the corresponding time point. During the prediction phase, the model takes the observable segment of the sequence as input and employs a recurrent prediction mechanism to progressively infer the activity state at the specified target time point. The VFGF framework comprises three core components: virtual-semantic preprocessing, recurrent sequence prediction, and target activity generation. These components work synergistically to refine semantic sequences and dynamically guide predictions, effectively tackling the challenges of dynamic scenarios and long-term predictions. The design and implementation of each component are detailed below, emphasizing their critical contributions to accurate egocentric activity prediction.

### 3.1. Visual Semantic Preprocessing

**Figure 2:**
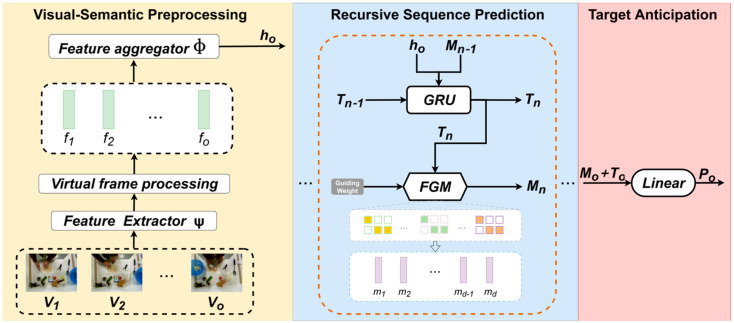
The VFGF for long-term egocentric activity prediction comprises three core components. The first component focuses on extracting visual features, virtual frames augmentation, and fusing sequential features to enrich the activity context. The second component employs a GRU for recursive prediction, complemented by a Transformer-based encoder–decoder architecture to process initial features and generate guiding features, which are subsequently used as input for the next time step in the GRU. The third component performs activity classification on the final predictions to yield anticipated outcomes for future action tasks.

The frames sequence $V\in R^{o\times d}$ derived from observable historical video segment serves as the sole input for model forecasting. To effectively encode these frame sequences for long-term activity prediction, this paper proposes a feature extraction and aggregation method enhanced by virtual frame augmentation (VFA). As illustrated in Figure 2, this approach extends the original feature sequence to smooth semantic transitions and integrates spatiotemporal feature extraction with recursive aggregation to construct rich contextual features, thereby establishing a robust foundation for accurate activity predictions.

To capture the spatiotemporal dynamics of the augmented sequence, a Temporal Segment Network (TSN) [37] is employed as the feature extractor $\psi \left(\cdot \right)$, to extract spatiotemporal features for each frame, Alternatively, other networks, such as I3D [38], can be utilized to achieve comparable feature extraction. The feature $f_{i}$ extracted from frame $v_{i}$ is represented as:

(1) $$f_{i}\;=\;\Psi \left(v_{i}\right)$$

where $f_{i}$ integrates local spatial information with global contextual associations. The TSN is selected for its superior performance in video spatiotemporal modeling, ensuring robust feature representations.

To mitigate semantic loss caused by rapid scene transitions or action changes, the technique of virtual frame augmentation is initially applied to the observable frame sequence. Given the feature $f_{i}$ of a frame, the similarity between the feature $f_{i+1}$ of an adjacent frame is computed to identify pairs with significant differences. A similarity vector $K=\left\{k_{1},k_{2},\;\;...,k_{o-1}\right\}\in R^{o-1}$ is defined to quantify the correlation between the feature of adjacent frame within the observable sequence. Cosine similarity is employed to calculate this vector K. For instance, the similarity $k_{i}$ between frames i and i+1, where $i\in \left(1,o-1\right)$, is computed as:

(2) $$k_{i}=\frac{f_{i}\;\cdot {\;f}_{i+1}}{\left\|f_{i}\|\times \|f_{i+1}\right\|}$$

where $\left(\cdot \right)$ denotes the dot product, computed as the sum of element-wise multiplications. Linear interpolation is used to generate virtual frames, as it efficiently produces spatially and temporally coherent sequences [39]. A threshold ε=0.8 is then defined as the minimum similarity required for smooth transitions between the features of adjacent frames, as validated in Section 4.5.2. The set of indices requiring insertion is $I_{i}=\left\{\begin{array}{cc} 1, & k_{i}<\varepsilon \\ 0, & else \end{array}\right.$. The total number of inserted feature is denoted as $\sum_{i=1}^{o-1}I_{i}$, resulting in the length of the extended sequence after the insertion of features being $L=\sum_{i=1}^{o-1}I_{i}+o$. The augmented sequence $F^{’}=\left\{f_{1}^{’},\;f_{2}^{’},\ldots ,\;f_{L}^{’}\right\}$ is generated via linear interpolation, and the calculation of the *t*-th frame $f_{t}^{’}\in R^{d}$ is as follows:

(3) $$f_{t}^{’}=\left\{\begin{array}{c} f_{x},\;if\;t=x+\sum_{i=1}^{x-1}I_{i},\;x\in \left(1,o\right)\\ p(f_{x})+(1-p)(f_{x+1}),\;if\;t=x+\sum_{i=1}^{x-2}I_{i},\;I_{x-1}=1,\;x\in \left(2,o\right) \end{array}\right.$$

where $p\in \left(0,1\right)$ represents the weight of the original frame $f_{i}$ in the virtual frame $f_{k}^{’}$, set to 0.5 by default. The augmented sequence $F^{’}$ is thus obtained.

**Figure 3:**
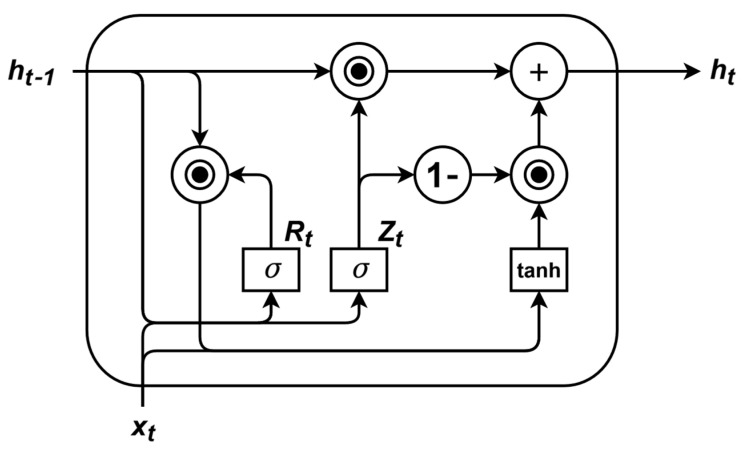
The GRU architecture. The current hidden state $h_{t}$ is determined jointly by the input $x_{t}$ at the current time step and the hidden state $h_{t-1}$ from the previous time step.

To aggregate the dynamic context of the feature sequence, a GRU is used as the recursive aggregator $\phi \left(\cdot \right)$, processing the feature sequence temporally up to the final time step:

(4) $$h_{o}=\phi \left(f_{1}^{’},{\;f}_{2}^{’},\ldots ,\;f_{L}^{’}\right)$$

where $h_{o}\in R^{d}$ represents the comprehensive feature over the entire observable time steps. The GRU’s gating mechanism effectively mitigates error accumulation, enhancing the stability of long-term predictions. Through these steps, this study constructs a model that efficiently integrates past video features, providing rich contextual information for subsequent activity prediction. The structure of the GRU is illustrated in Figure 3.

### 3.2. Recurrent Sequence Prediction

Building on the feature encoding enhanced by virtual frame augmentation, the recursive sequence prediction module iteratively processes contextual features to infer future activity states, serving as a cornerstone for long-term egocentric activity prediction. As illustrated in Figure 2 (Recursive Sequence Prediction), this module leverages the comprehensive feature representation $h_{o}$, generated by the preprocessing module, to iteratively predict the state at each subsequent time step until the target time step is reached.

For instance, at the target time step n, the model utilizes the guidance information $M_{n-1}$ from the previous time step and $h_{o}$ alongside to generate an initial prediction state $T_{n}\in R^{d}$ through a GRU layer:

(5) $$T_{n}=GRU\left(\left[h_{o},M_{n-1}\right],T_{n-1}\right)$$

At the onset of the recursive sequence, the initial values of $M_{n-1}$ and $T_{n-1}$ are set to $F_{o}\in R^{d}$, where $T_{n-1}$ denotes the hidden information from the previous time step of the GRU. However, as highlighted in the introduction, the initial prediction state often lacks sufficient accuracy in complex dynamic scenarios. Directly employing $T_{n}$ for subsequent recursive predictions can result in cumulative errors, significantly impairing long-term prediction performance.

To overcome this limitation, we introduce an FGM (Feature Guidance Module) that dynamically generates guiding semantic features to refine the recursive prediction process. By capturing latent associations between contextual features and target activities based on the current semantic environment, the FGM produces auxiliary features to steer the prediction direction (see Section 3.3 for implementation details). Unlike traditional recursive models, which heavily rely on observable information, the FGM effectively mitigates error accumulation, ensuring both stability and accuracy in predictions.

By integrating the initial prediction with the guiding features from the FGM, the model iteratively executes the prediction process until the target time step is reached, yielding the final activity state prediction. Owing to the optimized prediction mechanism, this module substantially enhances the robustness of long-term egocentric activity prediction, providing critical support for the framework’s overall performance breakthrough.

### 3.3. Feature Guidance Module

**Figure 4:**
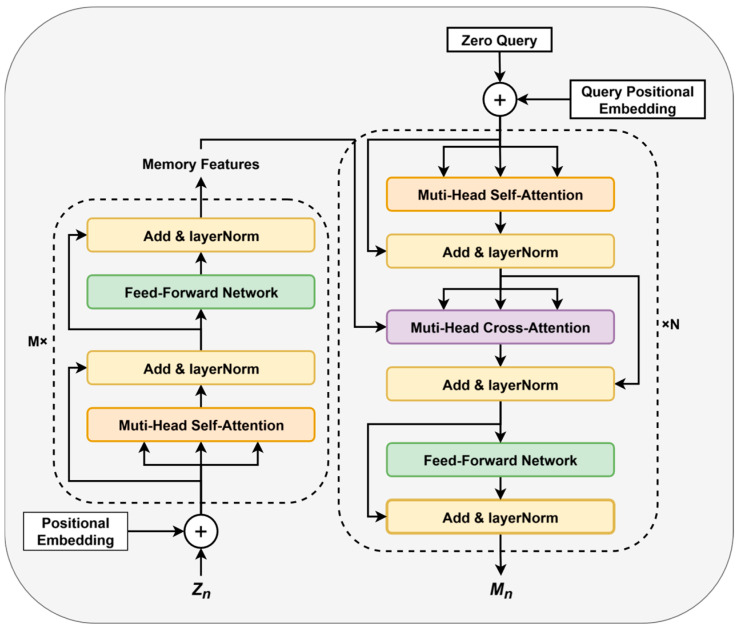
The FGM is composed of a Transformer-based encoder–decoder architecture, incorporating multi-head self-attention and multi-head cross-attention mechanisms. This design enables efficient capture of latent logical relationships within sequential data, providing effective guiding information for the GRU in recursive prediction tasks.

As discussed in Section 2.2, existing methods based on recursive prediction models suffer from significant performance degradation over extended prediction time steps due to error accumulation. To address the limitations and enhance the model’s ability to handle complex activity features in long-term activity prediction tasks, we propose the FGM, as shown in Figure 4.

The core objective of the FGM is to uncover latent relationships between features through pretraining and leverage the learned knowledge as a guiding mechanism during recursive training to assist the base recurrent network in effective prediction. The FGM is implemented using a transformer-based encoder–decoder framework, with the following details. First, the predictive features $T_{n}\in R^{d}$ are projected:

(6) $$Z_{n}=T_{n}W_{in}+b_{in}$$

where $W_{in}$ and $b_{in}$ are learnable parameters. Subsequently, learnable positional embeddings are added to the input sequence $Z_{n}\in R^{d}$,

(7) $$Z_{n}^{pos}=Z_{n}+P_{pos}$$

where $P_{pos}∽N\left(0,1\right)$.

The sequence $Z_{n}^{pos}\in R^{d}$ with positional encodings is then passed through M transformer blocks. Each transformer block consists of a multi-head self-attention mechanism, residual connections with layer normalization, and a feed-forward neural network. The multi-head self-attention mechanism incorporates learnable parameters, allowing different heads to capture distinct patterns during training. The propagation through each transformer block is defined as:

(8) $$X_{n}^{’}=LN\left(MHSA\left(Z_{n}^{pos}\right)+Z_{n}^{pos}\right)$$

(9) $$X_{n}=LN\left(FFN\left(X_{n}^{’}\right)+X_{n}^{’}\right)$$

The transformer preserves the dimensionality of the input feature sequence, ensuring that the output sequence maintains the same shape as the input, i.e., $X_{n}^{’}\in R^{d}$, $X_{n}\in R^{d}$.

Next, the decoding process begins. The decoder, which includes N transformer blocks, incorporates an additional multi-head cross-attention module to integrate the encoder’s output $X_{n}$ with the decoder’s query vectors $Q_{n}\in R^{d}$. The $Q_{n}$ are obtained similarly to $X_{n}$, as shown in Figure 4. The cross-attention is computed as:

(10) $$X_{n}^{cross}=Tranformer(X_{n},Q_{n})$$

Finally, $X_{n}^{cross}$ employs FNN and LN networks to produce the guiding features $M_{n}=\left\{m_{1},{\;m}_{2},\ldots ,\;m_{d}\right\}$ for the next frame. The FGM’s network weights are obtained through pretraining and remain fixed during the prediction process, relying solely on the pretrained knowledge to skillfully construct and introduce plausible future features. The experimental setup includes an encoder depth of M=2, a decoder depth of N=2, and a total of 8 attention heads. For more settings, please refer to the experimental section. This approach effectively leverages available contextual information, reducing the model’s strong dependence on observable data and mitigating error accumulation in recursive prediction sequences.

### 3.4. Training Objective Function

After completing recursive sequence prediction, the predictive representation $T_{o}$ and final guiding features $M_{o}$ are obtained at the target prediction time step o. The probability distribution of the target activity $P^{T}\in R^{N_{a}}$ at the final anticipated time step is computed using a linear layer with a softmax activation function, as shown in Equation (11):

(11) $$P^{T}=softmax\left(W_{T}[T_{o},M_{o}]+b_{T}\right)$$

Furthermore, the guiding features $M_{o}$ at the final anticipated time step are processed through a linear layer with a softmax activation function to compute prediction results $P^{M}\in R^{N_{a}}$, as shown:

(12) $$P^{M}=softmax\left(W_{M}M_{o}+b_{M}\right)$$

where $W_{T}\in R^{N_{a}\times 2d}$ and $W_{M}\in R^{N_{a}\times 2d}$ are learnable parameters, $N_{a}$ represents the number of activity categories, and d denotes the feature dimension of the current modality. Notably, the FGM parameters are fixed during training to ensure stability. Given the model parameters θ, the loss function is designed concisely, as shown in Equation (13):

(13) $$L\left(\theta \right)=L_{T}+\beta \cdot L_{M}$$

where $L_{C}$ and $L_{M}$ denote the standard cross-entropy losses for the target activity $T_{o}$ and guiding feature $M_{o}$ classifications. β is an adjustable weight coefficient. The FGM is pretrained using all frame information to capture intrinsic inter-frame relationships, enabling robust and high-precision guidance for long-term egocentric activity prediction.

## 4. Experimental Results

In this section, we describe the datasets and experimental protocols employed to evaluate the proposed method. Additionally, we present comparisons with state-of-the-art approaches and discuss the experimental results.

### 4.1. Dataset

To evaluate the effectiveness of VFGF, we conducted experiments on the EPIC-Kitchens dataset for long-term egocentric activity prediction. Collected from 32 participants performing unscripted activities in diverse kitchen environments, this dataset comprises 28,472 activity segments, encompassing 125 distinct actions (e.g., cutting, stirring), 331 specific noun categories (e.g., knife, onion), and 2513 unique activity classes (e.g., “cut onion”). Following the experimental protocol in [2], the public training set is split into 23,493 segments for training and 4979 for validation to assess model accuracy. The dataset, recorded using head-mounted cameras, offers a first-person perspective and a naturalistic setting that provide rich temporal and contextual information, making it ideal for evaluating long-term activity prediction. For EPIC-Kitchens, following [2], evaluation metrics include Top-5 accuracy as a class-agnostic measure of overall prediction performance and Mean Top-5 recall as a class-aware metric to address class imbalance, ensuring a robust foundation for validating the VFGF framework’s performance.

### 4.2. Compared Methods

To evaluate the superiority of our proposed Virtual Frame-Augmented Guided Forecasting (VFGF) method, we compared it with various existing approaches, including direct prediction methods from observed video clips (e.g., DMR [40], ATSN [2], MCE [41], SVM [42], ActionBanks [43], ED [44], FN [45], RL [46], EL [47]), recursive anticipation frameworks (e.g., RU-LSTM [12], LAI [48], SRL [14], HRO [16], SF-RULSTM [49]), and Transformer-based methods for prediction tasks (e.g., AVT [50], IAAM [18], VS-TransGRU:ES [21]). The baseline is a supervised backbone network. For fair comparison, the results were either sourced from the respective papers or reproduced using their published code. Experiments used a fixed random seed, reporting the average results, with all methods employing identical feature extraction and data splits for consistency.

### 4.3. Experimental Setting

The experiments were implemented by Pytorch 1.10 and conducted through one NVIDIA RTX 3090 GPU. Experiments were conducted on the EPIC-Kitchens dataset. The model processes fixed-length 1.5 s video clips as input, targeting activity progression prediction at multiple future time steps (0.25 s, 0.5 s, 0.75 s, 1 s, 1.25 s, 1.5 s, 1.75 s, and 2 s). The observation time step o is set to 6, providing six intervals of observed information, while the anticipation time step a is set to 8 to define the prediction horizon. For fair comparison, all methods use identical input features, with video segments sampled at 0.25 s intervals, selecting only the video frames corresponding to these time steps. Subsequently, RGB and optical flow features are extracted via a pretrained TSN model [2], each modality comprising 1024-dimensional spatiotemporal features. Additionally, 352-dimensional object features are derived from [12]. The features from all three modalities are normalized before being input into the prediction model. All models are followed by a linear layer at the final stage to output a probability distribution over 2513 actions as predictions. Results are sourced from the respective papers or reproduced using their published code, with experiments conducted using a fixed random seed and identical feature extraction and data splits for consistency. The FGM employs a transformer-based encoder–decoder architecture, utilizing self-attention and cross-attention mechanisms to capture long-range temporal and spatial dependencies, enhancing the model’s capability for complex activity prediction tasks. For feature extraction, multimodal data consistent with prior methods are used, with features adopted from [12] to ensure comparability. To enable fair comparisons with state-of-the-art methods, the TSN model, pretrained on a large-scale dataset as provided in [2], serves as the visual feature extractor. The recursive aggregator is implemented using a GRU, with its hidden state dimension aligned with the input visual feature dimension, and the classifier consists of a linear layer. In the virtual frame augmentation module, a similarity threshold of 0.3 is set to ensure smooth inter-frame transitions, determined through ablation studies reported in Section 4.5.2. The FGM is configured with 8 attention heads, and both the encoder and decoder consist of 2 transformer blocks each. Model parameters are optimized using the stochastic gradient descent optimizer with a learning rate of 0.05, momentum of 0.9, weight decay of $1\times 10^{-5}$, and batch size of 128, β of 0.1. The FGM is pretrained on the EPIC-Kitchens dataset with the same train-validation split as VFGF. Pretraining runs for 100 epochs with cross-entropy loss, using early stopping after 10 epochs of no validation loss improvement and a 0.05 dropout rate to prevent overfitting, ensuring robust feature extraction for VFGF’s long-term prediction.

VFGF processes multimodal inputs (RGB, optical flow, and object features) to enhance prediction robustness. Each modality is handled by pretrained branch models, with optimal parameters detailed in our open-source code. Specifically, RGB and optical flow features are extracted using a pretrained TSN model [2], while object features are derived from [12]. Each modality employs a pretrained FGM, followed by VFGF training. Predictions from the three modalities are fused via a three-layer MLP (MATT, dropout rate 0.8) to generate a unified multimodal representation, optimized using cross-entropy loss over 50 epochs.

### 4.4. Comparison with Other Methods

Table 1 presents the comparative performance of the proposed VFGF against state-of-the-art methods on the EPIC-Kitchens validation set. To comprehensively highlight VFGF’s advantages, we report performance under multimodal inputs (RGB, FLOW, OBJ). In addition to evaluating Top-5 action accuracy across eight anticipation time steps (0.25 s to 2.0 s), we compare Top-5 accuracy for actions, verbs, and nouns, as well as Mean Top-5 recall (class-agnostic and class-aware) at a 1 s anticipation time. Bold and underlined values indicate the best and second best results, respectively. The experimental results demonstrate that VFGF outperforms competing methods across most evaluated anticipation times. Specifically, at a 1 s anticipation time, VFGF achieves a Top-5 action accuracy of 38.53%, surpassing the previous best recursive models, SF-RULSTM [48] and HRO [16], by 2.44% and 1.11%, respectively, and outperforming the Transformer-only AVT [50] method by 0.93%. Across all time steps, VFGF achieves an average improvement of 0.89% over the hybrid Transformer-GRU model VS-TransGRU:ES [21], with a notable 1.56% average gain in short-term predictions (0.25 s to 1.0 s).

For a summary, the comparative analysis reveals that VFGF consistently outperforms other models due to its reduced reliance on strongly coupled observable information compared to traditional models like SF-RULSTM and HRO, which are based on LSTM [51] and GRU [22]. Compared to the Transformer-only AVT method, VFGF also demonstrates significant advantages, as the memory capabilities inherent to recursive units are better suited for long-term prediction tasks. Furthermore, VFGF maintains a performance edge over VS-TransGRU:ES, which combines Transformer and GRU, underscoring the importance of well-designed integration to leverage the strengths of different modules for optimal long-term prediction performance.

### 4.5. Ablation Experiments

Ablation experiments were conducted on the RGB modality of the EPIC-Kitchens [2] dataset, leveraging its rich activity scenarios and diverse multimodal information to comprehensively evaluate performance under different feature combinations.

#### 4.5.1. Module Effectiveness

**Baseline**: The baseline model (denoted as “Baseline” in Table 2) employs the same observed information encoding as VFGF. In the recursive sequence prediction phase, only a single GRU layer is used to predict feature representations at each anticipation time step, followed by a fully connected layer with a softmax activation function for target activity prediction. The Adam optimizer (learning rate of 0.001) and cross-entropy loss over 100 epochs, with early stopping applied to prevent overfitting. According to Table 2, the baseline model achieves a Top-5 action accuracy of 29.18% and a Top-1 action accuracy of 13.47% at a 1 s anticipation time, indicating substantial room for improvement in predictive performance.

**Baseline + VFA**: Incorporating the VFA module (denoted as “+VFA” in Table 2) improves the Top-5 action accuracy from 29.18% to 30.01% at a 1 s anticipation time, demonstrating the effectiveness of VFA. Across all anticipation time steps (0.25 s to 2.0 s), VFA consistently enhances performance in both Top-5 and Top-1 action accuracy results. This improvement stems from VFA’s ability to insert virtual frames, mitigating differences between consecutive frames and smoothing transitions, thereby enriching the semantic quality of observable segments.

**Baseline + FGM**: Integrating the FGM (denoted as “+FGM” in Table 2), pretrained as described in Section 4.3, the FGM loads fixed parameters, receives GRU outputs, and generates guiding features for the next time step, assisting the GRU in long-term prediction tasks. This combination yields a 2.36% improvement in Top-5 action accuracy over the baseline at a 1 s anticipation time. Notably, performance gains are observed across various time steps, with more significant improvements in short-term predictions, such as a 3.73% gain at 0.25 s compared to a 2.53% gain at 2.0 s. This trend is attributed to the increasing error accumulation in the GRU over longer time steps, which degrades the initial prediction quality and leads to less accurate guiding features from the FGM, thereby mutually affecting performance.

**Baseline + VFA & FGM**: Combining VFA and the FGM (denoted as “Baseline + VFA & FGM” in Table 2) results in performance superior to using either module alone. The addition of VFA to “Baseline + FGM” yields more significant improvements than VFA alone, as VFA enhances the quality of observable information, improving GRU prediction accuracy and providing more precise input features for the FGM’s queries.

**Baseline + Combination of All Components**: As shown in the final row of Table 2, incorporating classification loss on the guiding features generated by the FGM further enhances VFGF’s overall prediction performance, significantly improving both Top-5 and Top-1 predictive accuracy. Since FGM parameters are fixed during training, computing loss on its guiding features encourages the GRU to learn more effective representations, providing the FGM with more accurate initial features.

#### 4.5.2. Selection of Virtual Frame Threshold ε

**Figure 5:**
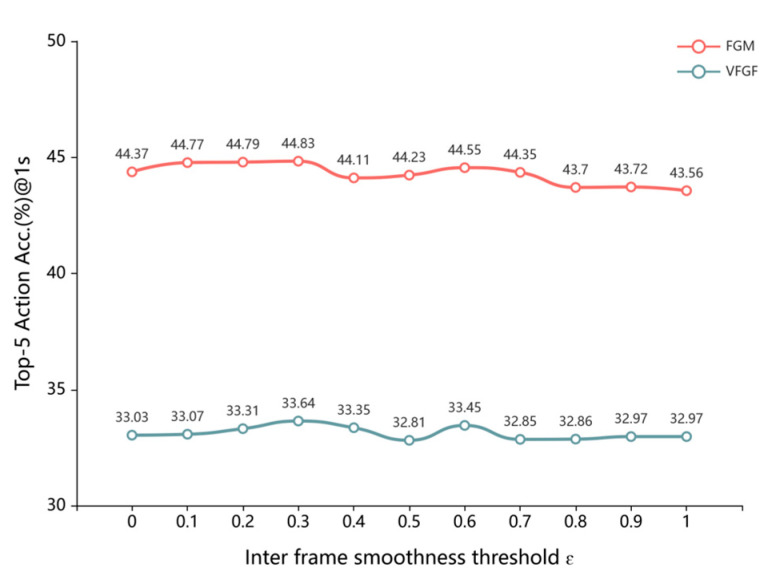
Virtual frame filling threshold selection experiment on EPIC Kitchen validation set (RGB).

To determine the optimal similarity threshold ε for virtual frame insertion, as discussed in Section 3.1, we conducted a grid search, with results illustrated in Figure 5. The results compare the performance of the FGM and VFGF with VFA at various threshold values, including a case without VFA. Both models exhibit similar performance trends across thresholds ranging from 0 to 1. Notably, at thresholds between 0.1 and 0.5, VFA yields significant performance improvements. However, an increase in the threshold beyond 0.5 leads to performance degradation due to excessive insertion of similar frames, introducing redundant information that hinders the generation of high-quality comprehensive feature representations, ultimately reducing prediction accuracy.

### 4.6. Parameters and FLOPs

The VFGF model demonstrates a compelling balance between predictive performance and computational efficiency, as presented in Table 3. With a parameter count of 25.13 M and computational complexity of 74.41 G FLOPs, VFGF achieves a Top-5 accuracy of 33.64%, outperforming alternative models such as AVT (382.81 M parameters, 8573.46 G FLOPs, 28.10% accuracy) and SRL (29.31 M parameters, 2115.91 G FLOPs, 31.68% accuracy). This efficiency arises from the optimized graph-based feature fusion mechanism, which makes VFGF well suited for resource-constrained environments, including edge devices used in real-time video detection tasks. However, VFGF’s parameter count remains higher than that of lighter models like TransGRU (15.96 M parameters, 793.47 G FLOPs, 32.36% Top-5 accuracy), potentially limiting its applicability in extremely resource-scarce scenarios. Moreover, the simplified graph-based fusion may compromise robustness under highly dynamic or noisy inputs, suggesting opportunities for further refinement through advanced regularization or hybrid architectural designs.

### 4.7. Generalization Experiments

**Figure 6:**
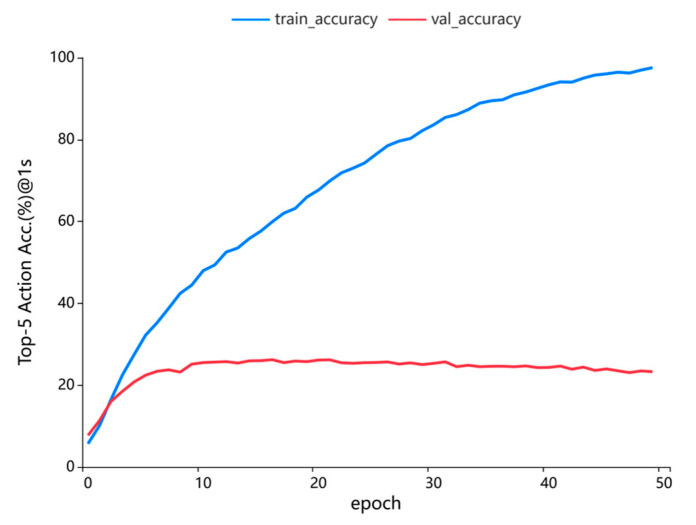
Convergence of VFGF generalization on the EPIC-Kitchens dataset, with training accuracy (blue) and validation accuracy (red) over epochs.

To rigorously evaluate the generalization capability of the Virtual Frame-Augmented Guided Forecasting (VFGF) framework, comprehensive experiments were conducted on the EPIC-Kitchens dataset using 5-fold cross-validation to ensure robust and reliable train-validation splits, with a training-to-validation ratio of 2:8. The Feature Guidance Module (FGM) was pretrained for 50 epochs using stochastic gradient descent (SGD) with a learning rate of 0.05, momentum of 0.9, weight decay of 0.0001, and a batch size of 128. Subsequently, the VFGF framework was trained for an additional 50 epochs using cross-entropy loss to optimize its predictive performance. Figure 6 illustrates the median performance across five experimental runs, with the blue line representing the convergence of training accuracy and the red line indicating the convergence of validation accuracy. The VFGF model achieved convergence on the validation set by the 16th epoch, attaining a Top-5 accuracy of 26.21%. However, the training accuracy curve indicates overfitting beyond this point, with training performance continuing to improve while validation performance stabilizes or declines. This rapid convergence and competitive Top-5 accuracy underscore the efficacy of virtual frame augmentation and the FGM in enhancing model robustness. These advancements enable VFGF to deliver consistent and high-quality performance across diverse egocentric scenarios in the EPIC-Kitchens dataset, demonstrating its strong generalization potential.

## 5. Discussion

**Figure 7:**
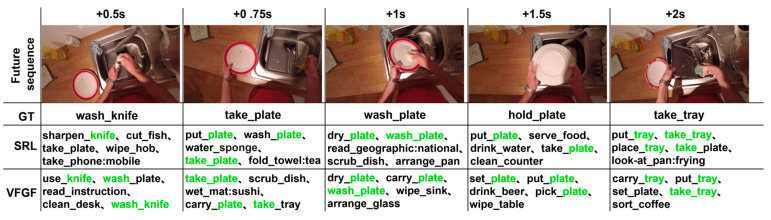
Top-5 prediction accuracy comparison between VFGF and SRL on the EPIC-Kitchens dataset, with correct predictions highlighted in green.

The Virtual Frame-Augmented Guided Forecasting (VFGF) framework achieves state-of-the-art performance on the EPIC-Kitchens dataset, demonstrating the effectiveness of virtual frame augmentation and the Feature Guidance Module (FGM). Compared to the Self-Regulated Learning (SRL) framework [14], VFGF produces Top-5 predictions that align more closely with ground truth, as shown in Figure 7, where correct predictions for “take_tray” at +0.75 s match the 2 s ground truth (highlighted in green), underscoring FGM’s role in enhancing semantic guidance. VFGF outperforms SRL in short-term predictions but, like SRL, faces performance degradation at +1.5 s due to error accumulation. Against TransGRU [16], a Transformer-based model, VFGF offers competitive accuracy with lower computational complexity due to its hybrid GRU-Transformer architecture. Unlike traditional recursive methods [12,13,14,15], which rely heavily on local data and suffer from error accumulation, or other Transformer-based approaches [16,17,18,19,20,21], VFGF leverages virtual frame augmentation for improved semantic continuity and combines GRU’s localized temporal processing with Transformers’ global dependency modeling, achieving a balance of efficiency and robustness motivated by the need to address error accumulation and computational demands in dynamic egocentric scenarios.

VFGF’s precise short-term predictions enable applications like human–robot interaction and video surveillance, where timely action forecasting is critical, and its efficient GRU-Transformer design enhances suitability for resource-constrained devices, such as wearables. Its robust generalization on EPIC-Kitchens suggests potential for smart homes or assistive technologies. However, VFGF’s large parameter count, akin to other Transformer-based models, poses challenges for edge device deployment. Additionally, performance degradation at longer horizons (e.g., +1.5 s) due to error accumulation limits long-term prediction reliability, and its effectiveness may wane on smaller datasets with limited data diversity. Future work could explore Transformer pruning or knowledge distillation to create lightweight models [52] and develop adaptive multimodal fusion to reduce error accumulation (e.g., combining text and audio [53,54,55,56]), thereby improving scalability and long-term action predictions.

## 6. Conclusions

This study presents the VFGF framework for long-term egocentric activity prediction, evaluated on the EPIC-Kitchens dataset using multimodal inputs (RGB, FLOW and OBJ). The experimental results demonstrate that VFGF achieves superior performance, particularly in short-term predictions, while maintaining robust Top-5 action accuracy across multiple anticipation intervals ranging from 0.25 s to 2.0 s. Ablation studies focused on the RGB modality confirm the effectiveness of both the VFA and FGM. The VFA improves the quality of observable sequences by addressing semantic gaps caused by abrupt changes in activity or environment. Meanwhile, the FGM enhances prediction accuracy by supplying semantically relevant guiding features during the recursive prediction process. Their combined use, along with a classification loss applied to FGM-generated features, significantly strengthens GRU-based learning and improves long-term inference capabilities. Collectively, these components demonstrate the innovative integration of pretraining, virtual frame augmentation, and a hybrid GRU-Transformer architecture, offering a context-aware solution well-aligned with real-world activity dynamics. The VFGF framework sets a new benchmark in egocentric activity forecasting by improving semantic continuity and predictive stability.

Future work will focus on extending VFGF to incorporate additional modalities such as depth, audio, and inertial sensor data, enriching the semantic representation of egocentric contexts. To integrate these heterogeneous inputs, we will explore multimodal fusion strategies like cross-modal transformers and co-attention mechanisms to improve robustness and adaptability in complex activity scenes. Further improvements will include developing adaptive VFA thresholds, advanced pretraining strategies such as self-supervised or contrastive learning for the FGM, and lightweight architectures for real-time inference on resource-constrained devices. Finally, comprehensive evaluations across broader datasets will validate the framework’s generalizability for applications in human–robot interaction and wearable computing.

## Figures and Tables

**Table 1:** Anticipation Results of Different Methods on EPIC-Kitchens Dataset.

Model	Top-5 Activity Accuracy (%) @ Different $\tau_{a}$(s)								Top-5 Acc. (%) @ 1s			M. Top-5 Rec. (%) @ 1s		
	2	1.75	1.5	1.25	1	0.75	0.5	0.25	Verb	Noun	Act.	Verb	Noun	Act.
DMR [40]	/	/	/	/	16.86	/	/	/	73.66	29.99	16.86	24.50	20.89	03.23
ATSN [2]	/	/	/	/	16.29	/	/	/	77.30	39.93	16.29	33.08	32.77	07.60
MCE [41]	/	/	/	/	26.11	/	/	/	73.35	38.86	26.11	34.62	32.59	06.50
SVM [42]	/	/	/	/	25.42	/	/	/	72.70	38.41	25.42	41.90	34.69	05.32
ActionBanks [43]	/	/	/	/	28.60						28.60			
ED [44]	21.53	22.22	23.20	24.78	25.75	26.69	27.66	29.74	75.46	42.96	25.75	41.77	42.59	10.97
FN [45]	23.47	24.07	24.68	25.66	26.27	26.87	27.88	28.96	74.84	40.87	26.27	35.30	37.77	06.64
RL [46]	25.95	26.49	27.15	28.48	29.61	30.81	31.86	32.84	76.79	44.53	29.61	40.80	40.87	10.64
EL [47]	24.68	25.68	26.41	27.35	28.56	30.27	31.50	33.55	75.66	43.72	28.56	38.70	40.32	08.62
RU-LSTM [12]	29.44	30.71	32.33	33.41	35.32	36.34	37.37	38.98	79.55	51.79	35.32	43.72	49.90	15.10
LAI [48]	/	/	32.50	33.60	35.60	36.70	38.50	39.40	80.00	52.80	35.60	/	/	/
SRL [14]	30.15	31.28	32.36	34.05	35.52	36.77	38.60	40.49	/	/	35.52	/	/	/
HRO [16]	31.30	32.67	34.26	35.87	37.42	38.36	39.89	42.36	**81.53**	54.51	37.42	45.16	51.78	17.50
SF-RULSTM [49]	30.58	/	32.83	/	36.09	/	37.87	/	/	/	36.09	/	/	/
AVT [50]	/	/	/	/	37.60	/	/	/	/	/	37.60	/	/	/
IAAM [18]	/	/	/	/	35.45	/	/	/	80.26	53.10	35.45	44.84	53.43	17.14
VS-TransGRU:ES [21]	32.13	33.57	35.10	36.69	**38.78**	39.12	39.87	40.95	79.78	55.69	**38.78**	46.23	52.91	**18.29**
VFGF (Ours)	**32.54**	**33.72**	**35.27**	**36.84**	38.53	**40.58**	**41.75**	**44.11**	80.75	**56.36**	38.53	**47.82**	**57.75**	17.91

The bold indicates the best performance, while the underline denotes the second-best.

**Table 2:** The effectiveness of each component on EPIC-Kitchens validation set (RGB).

Exp.	VFA	FGM	$L_{T}$ + $L_{M}$	Top-5 Accuracy (%) at Different $\tau_{a}$ (s)						Top-1 Accuracy (%) at Different $\tau_{a}$ (s)					
				2	1.75	1.5	1	0.5	0.25	2	1.75	1.5	1	0.5	0.25
baseline	-	-	-	24.32	25.06	26.29	29.18	31.42	33.75	10.52	11.15	11.95	13.47	16.19	18.43
+VFA	√	-	-	24.76	25.51	27.04	30.01	32.22	34.43	10.58	11.29	12.13	14.18	17.56	19.16
+FGM	-	√	-	26.85	27.36	28.29	31.54	34.60	37.48	11.71	12.13	13.37	14.58	18.62	20.92
+VFA&FGM	√	√	-	27.66	28.60	29.79	32.57	36.02	38.91	12.57	13.09	14.20	16.25	18.91	21.61
+All components	√	√	√	28.52	29.38	30.06	33.64	36.77	39.26	13.09	13.37	14.32	16.29	19.20	22.72

The symbol “√” indicates that the corresponding module is utilized, while “-” denotes that the module is not used.

**Table 3:** The results of parameters and FLOPs.

Models	$Parameters\;({\times 10}^{6})$	$FLOPs\;({\times 10}^{6})$	Top−5 Acc. (%) @1s
RULSTM [12]	19.37	1153.89	30.83
SRL [14]	29.31	2115.91	31.68
AVT [50]	382.81	8573.46	28.10
VS-TransGRU:ES [21]	15.96	793.47	32.36
VFGF	25.13	74.41	33.64

## Data Availability

The datasets used in this study are available at https://github.com/fpv-iplab/rulstm (accessed on 1 June 2025). The source code of VFGF will be released after acceptance at https://github.com/MCLXD/VFGF (accessed on 7 September 2025).

## Abbreviations

VFGF	Virtual Frame-Augmented Guided Prediction Framework
LTA	Long-term Action Anticipation
FGM	Feature Guidance Module
GRU	Gated Recurrent Unit
VR	Virtual Reality
RGB	Red, Green, Blue
LSTM	Long Short-Term Memory
H3M	Hierarchical Multitask MLP Mixer Model
I-CVAE	Intention-Conditioned Variational Autoencoder
CDFSL	Cross-Domain Few-Shot Learning
TSN	Temporal Segment Network
VFA	Virtual Frame Augmentation
